# Structural Differences in Insular Cortex Reflect Vicarious Injustice Sensitivity

**DOI:** 10.1371/journal.pone.0167538

**Published:** 2016-12-08

**Authors:** Thomas Baumgartner, Anne Saulin, Grit Hein, Daria Knoch

**Affiliations:** Department of Social Psychology and Social Neuroscience, Institute of Psychology, University of Bern, Bern, Switzerland; University of Zurich, SWITZERLAND

## Abstract

Sensitivity to injustice inflicted on others is a strong motivator of human social behavior. There are, however, enormous individual differences in vicarious injustice sensitivity. Some people are strongly affected when witnessing injustice, while others barely notice it, but the factors behind this heterogeneity are poorly understood. Here we examine the neuroanatomical basis of these differences using voxel-based morphometry and Freesurfer image analysis suite. Whole brain corrected analyses show that a person’s propensity to be vicariously affected by injustice to others is reflected by the gray matter volume and thickness of the bilateral mid insular cortex. The larger a person’s gray matter volume and thickness of the mid insula, the higher that person’s sensitivity to injustice experienced by others. These findings show that the individual neuroanatomy of the mid insular cortex captures a person’s predisposition to be vicariously affected by injustice, and thus adds a novel aspect to previous functional work that has linked this region to the processing of transient vicarious states.

## Introduction

Humans commit to political, societal, and environmental causes because they are affected by injustice inflicted on others. However, there is enormous heterogeneity in how sensitive people are to vicariously experienced injustice. Some individuals are outraged when witnessing injustice to others, while others watch injustice callously [1]. Previous studies have documented the individual heterogeneity in vicarious injustice sensitivity [2,3], and highlighted its strong predictive power for actual social outcomes. For example, vicarious injustice sensitivity is related to the propensity of altruistic sharing [4–6], and altruistic punishment [7], and has an impact on moral judgments [8]. This evidence demonstrates that understanding heterogeneity in vicarious injustice sensitivity is essential for understanding individual differences in social outcomes. However, so far, the sources of individual heterogeneity in vicarious injustice sensitivity remain unclear.

Recent applications of brain morphometry indicate that individual differences in brain structure can be useful in understanding individual differences in personality traits [9–11], social behaviors [12–15], and skills [16–19]. We therefore conjectured that variables reflecting relatively stable neuroanatomical individual differences, such as gray matter (GM) volume and cortical thickness, may help to predict individual differences in vicarioius injustice sensitivity.

Previous brain imaging studies have studied the functional neural responses during the vicarious experience of injustice to others, for example when others receive an unjustly small share of an available monetary amount [20,21]. According to their results, observing injustice to others is associated with neural activation in the mid and anterior insular cortex [20,21], in line with a large body of work that has linked this region to other vicarious experiences such as pain [22,23], embarrassment [24,25], or ostracism [26,27]. Based on these findings, we hypothesized that the structural differences of the mid and anterior insular cortex may provide a plausible neuroanatomical basis for situation- and context-independent individual differences in vicarious injustice sensitivity.

To test this assumption, participants imagined and rated scenarios in which others were subjected to injustice, taken from the well-established Justice Sensitivity Scale [28]. In an independent MRI session, we acquired structural images (T1-weighted MRI scans), which were analyzed with voxel-based morphometry (VBM), a whole-brain technique capable of discovering subtle, regionally specific changes in gray matter volume and Freesurfer image analysis suite, a technique that allows to disentangle the effects of cortical thickness and cortical surface.

## Results

**Fig 1:**
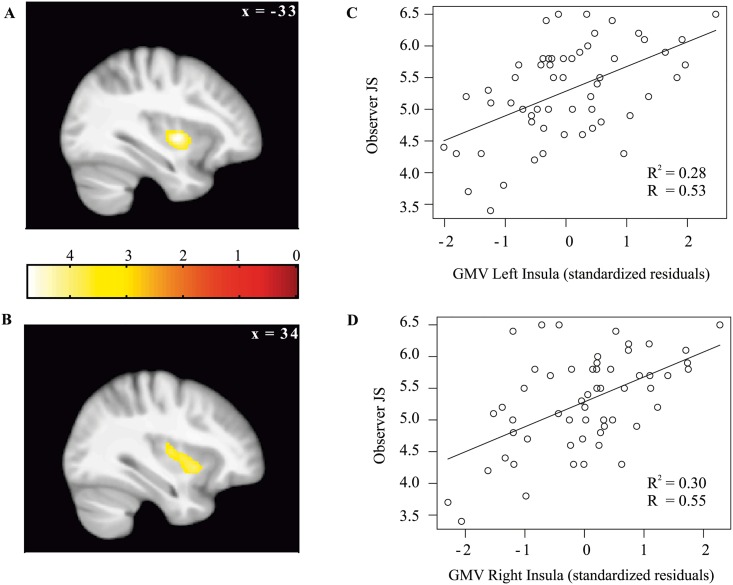
Observer Justice Sensitivity is positively associated with insular gray matter volume. The gray matter volume (GMV) is adjusted for age and brain-size. (A) Effect shown in sagittal view of the left insula (at p < 0.05, FWE-corrected for the whole brain, for display purpose depicted at p < 0.001 uncorrected). (C) Scatter plot of Observer Justice Sensitivity (JS) against the z-standardized GMV of the significant cluster in the left insula. (B) Same as (A) for right insula. (D) Same as (C) for right insula.

To determine regions whose gray matter volume is associated with the individual injustice sensitivity, the individual vicarious injustice ratings were regressed against the gray matter volume images using VBM. Age and brain size were included as control variables [29,30]. Family-wise error (FWE) whole-brain cluster correction was used to control for multiple comparisons. In line with our assumptions, the results showed a significant positive correlation between the gray matter volume of the bilateral mid insular cortex and the vicarious injustice sensitivity ratings, left insula, center-coordinate, x = -33, y = -6, z = -5; p < 0.05, corrected, r = 0.528, R^2^ = 0.28; right insula, center-coordinate, x = 34, y = 10, z = 17, p < 0.05, corrected, r = 0.548, R^2^ = 0.30 (Fig 1A and 1B). The larger the gray matter volume of the bilateral insula the greater the participant’s sensitivity to injustice inflicted on others (Fig 1C and 1D).

The correlation analysis did not reveal any other significant neural effects, i.e., the mid insular cortex was the only brain structure that was significantly linked to vicarious injustice sensitivity.

To test for the impact of gender, we conducted the same analysis with gender as additional co-variate. The results confirmed the significant positive correlations between the gray matter volume of the bilateral mid insular cortex and the vicarious injustice sensitivity ratings (left insula, p < 0.05, corrected, r = 0.516, R^2^ = 0.27; right insula, p < 0.05, corrected, r = 0.538, R^2^ = 0.29). This indicates the validity of our findings irrespective of subjects’ gender.

In order to obtain a more fine-grained understanding of the underlying structural differences driving the relationship between insular gray matter volume and vicarious injustice sensitivity, we additionally performed a vertex-based structural analysis with the Freesurfer image analysis suite (see material and methods for details). Freesurfer allows for looking at the two components of gray matter volume separately, i.e. cortical thickness and cortical surface. This analysis is thus able to provide evidence of whether the differences observed in gray matter volume of the insular cortex are due to differences in cortical thickness, cortical surface, or a combination of both. In order to answer this question, we conducted linear regression analyses with either the cortical thickness or the cortical surface component of the insular cortex as the dependent variable and the vicarious injustice sensitivity ratings as independent variable, controlling for age and intracranial brain volume. Results showed that only cortical thickness was positively associated with the Observer Justice Sensitivity (right Insula: r = 0.392, p = 0.003; left Insula: r = 0.366, p = 0.007), whereas cortical surface was not (right Insula: r = 0.137, p = 0.323; left Insula: r = -0.098, p = 0.482).

An additional analysis that included gender as co-variate revealed comparable results for cortical thickness (right Insula: r = 0.394, p = 0.003; left Insula: r = 0.366, p = 0.007) and surface area (right Insula: r = 0.134, p = 0.340; left Insula, r = -0.103, p = 0.464), indicating that the results are not altered by subjects’ gender.

## Discussion

These findings demonstrate a link between neuroanatomical brain structure and vicarious injustice sensitivity. The larger the GM volume and thickness of a person’s bilateral mid insular cortex, the higher this person’s propensity to respond sensitively to injustice inflicted on others. Previous studies have shown that vicarious injustice sensitivity varies enormously among individuals, and is relatively stable across time within a person [2,3], without accounting for this heterogeneity and temporal stability. The present study reveals inter-individual variation in the gray matter volume and thickness of the insular cortex, which, as we show here, can account for the individual heterogeneity in vicarious injustice sensitivity.

In contrast to previous studies that assessed neural responses “online” when subjects actually witnessed situational and context-dependent injustice, our study uses an “offline” measure of vicarious injustice sensitivity that is unadulterated by context or task demands [16]. Our finding that the neuroanatomy of the mid insular cortex reflects this “offline” measure thus captures a person’s predisposition to vicariously respond to injustice inflicted on others.

Based on anatomical connectivity studies it is well established that the insula is the first cortical target of ascending interoceptive [31] and viscerosensory [32] inputs. Given this evidence it has long been considered to be important for the generation of subjective feelings that guide decision making [33]. Remarkably, such subjective feelings are not only generated by first-hand inputs, but also by sensory and emotional experiences observed in others [20–22,24–27]. Aggregating these results, recent models propose that the mid and anterior insular cortex plays a central role in assigning relative salience to direct and vicarious inputs, and, as a result, determines the emotional and behavioral relevance of first-hand and vicarious experiences [34].

In the context of our findings, this might indicate that individuals with a larger mid insular cortex experience unjust treatment to others more saliently, which leads to a higher degree of vicarious injustice sensitivity. This interpretation is supported by the results of functional MRI studies that show an increase of functional neural response in this region in response to witnessed injustice [20,21]. The finding that previous functional “online” measures and our structural “offline” measures converge in the same region might suggest that the mid insular cortex integrates the state (captured by functional activation) and trait (captured by brain structure) dimensions of vicarious injustice sensitivity.

In this case, a person’s individual vicarious injustice sensitivity trait, reflected by the mid insula volume and thickness, should predict the magnitude of the “online” functional response during actual vicarious injustice experiences. Vice versa, inspired by studies that show the impact of long-term social factors on neuroplasticity [35,36], it is also conceivable that an enduring state of vicarious injustice experiences alters a person’s injustice sensitivity trait, captured by the mid insula GM volume and thickness.

Our results show that cortical thickness, but not cortical surface area is associated with a person’s injustice sensitivity trait. Cortical thickness and surface area are considered independent neuroanatomical traits that are likely to be influenced by different factors during brain development [37] and have different origins [38]. While cortical surface area increases during late fetal development due to cortical folding, cortical thickness alters dynamically across the entire life span as a consequence of experience, training and disease. This implies that the individual differences in cortical thickness of the insular cortex we observed might be less rooted in early cortical development, but rather be driven by different life experiences (e.g. experiences of injustice) and moreover, could be altered by training (e.g. empathy training).

It is noteworthy that previous research has associated the mid and anterior insular cortex to the processing of empathy [22], i.e., the affective sharing and understanding of both pleasant and unpleasant emotions [39]. Our finding that the neuroanatomy of the same structure predicts individual differences in vicarious injustice sensitivity is in line with recent work that has linked trait empathy to a person’s willingness to punish witnessed injustice [40], and supports models that assume a close relationship between empathy, justice and morality [41,42].

In sum, our study provides the neuroanatomical basis for vicarious injustice sensitivity, and thereby an account for its enormous individual heterogeneity. Based on recent models [34] we propose that the gray matter volume and thickness of the mid insular cortex might reflect the relative salience assigned to injustice inflicted on others, and thus determines a person’s predisposition for vicarious injustice sensitivity.

## Materials and Methods

### Participants

56 healthy subjects were studied (mean age ± S.D. = 22.3 ± 3.47 years, 26 females, 30 males). Participants gave written informed consent prior to participating in the study. The Ethics Committee of Basel (Ethikkommission beider Basel, EKBB) approved the study, which was conducted according to the principles expressed in the Declaration of Helsinki. The methods were carried out in accordance with approved guidelines. No subject had a history of psychiatric illness or neurological disorders. Subjects filled out the questionnaire and received 40 Swiss Francs (CHF 40; CHF 1 = about $1 U.S.) for participation. MRI scans were obtained in a separate session.

### Materials

Subjects were presented with ten items, and instructed to rate their response to injustice inflicted on others on a 7-point Likert scale (e.g., *I am upset when someone is undeservingly worse off than others*. *It takes me a long time to forget when someone else has to fix others’ carelessness*.). The material was taken from the observer perspective subscale of the Justice Sensitivity Scale [2], a highly reliable and consistent measure of individual differences in vicarious injustice sensitivity reliability = .97; trait consistency = .60 [43].

### MRI data acquisition

Measurements were performed on a 3 T whole body MR system (Magnetom Verio, Siemens Healthcare, Germany) equipped with a standard twelve-channel head coil. Anatomical images were acquired with a 3D magnetization prepared rapid gradient-echo (MPRAGE) sequence. The following acquisition parameters were used: TR (repetition time) = 2000 ms, TE (echo time) = 3.4 ms, TI (inversion time) = 1000 ms, flip angle = 8°, FOV (field of view) = 25.6 cm, acquisition matrix = 256 × 256 × 176, voxelsize: 1 mm × 1 mm × 1 mm. A sagittal volume covering the entire brain was acquired in 7.5 min.

### Neuroimaging data processing and statistical analyses

We applied in this study the same anatomical analyses as described in detail previously in a recent study on intergroup bias [13]. Anatomical brain images of 56 individuals were analyzed using voxel-based morphometry version 8 (VBM 8) implemented in statistical parametrical mapping version 8 (SPM 8). VBM 8 is documented and freely available online (http://dbm.neuro.uni-jena.de/vbm/). The method is based on high-resolution structural three-dimensional magnetic resonance images, registered in standard space, and is designed to find significant regional differences throughout the brain by applying voxelwise statistics within the context of Gaussian random fields [44]. Preprocessing of the data involved spatial normalization, segmentation into gray matter (GM), white matter (WM), and cerebrospinal fluid (CSF), modulation, and spatial smoothing with a Gaussian kernel (full width at half maximum = 8, [44,45]. In detail, the segmentation approach is based on an adaptive Maximum a Posterior (MAP) technique without the need for a priori information of tissue probabilities. It uses a Partial Volume Estimation (PVE) with a simplified mixed model of at most two tissue types, and applies a classical Markov Random Field (MRF) approach, which incorporates spatial prior information of adjacent voxels into the segmentation estimation. Finally, the modulation option we used during preprocessing multiplies the voxel values by the non-linear component derived from the spatial normalization, producing tissue volumes that are already corrected for individual brain size.

A linear regression analysis was performed on the smoothed gray matter volume images in SPM 8 to determine regions in which gray matter volume is associated with Observer Justice Sensitivity. Age was included in the design matrix as covariate of no interest to model and thus regress out any effects correlated with this factor. Note that the gray matter volume maps are already corrected for individual brain size; inclusion of individual brain size as an additional covariate was thus not necessary. We used a primary cluster-forming threshold of p < 0.001 for the whole brain volume as the criterion to detect voxels with a significant correlation with Justice Sensitivity scores. Clusters with a significant Family-Wise-Error (FWE) correction (p < 0.05) on a cluster level are reported.

In order to corroborate the findings of the VBM 8 analysis and to obtain a more fine-grained understanding of the underlying structural differences driving the relationship between insular gray matter volume and Observer Justice Sensitivity, we additionally performed a surface-based structural analysis with the Freesurfer image analysis suite, which is documented and freely available for download online (http://surfer.nmr.mgh.harvard.edu/). The technical details of these procedures are described in prior publications [13, 46–50]. Briefly, T1-weighted MRI images volumes were processed in a fully automated fashion using a cortical surface-based reconstruction that ultimately provides measurement of cortical thickness and surface area throughout the cortical mantle for each individual participant. In order to examine whether thickness or surface area, or a combination of both, drives the relationship between Observer Injustice Sensitivity and insular gray matter volume, we extracted the average thickness and surface area in this brain region. We then conducted linear regression analyses with the Observer Justice Sensitivity score as the independent variable and either the cortical thickness or surface area as the dependent variable, controlling for the same co-variates used in the VBM 8 analysis (age, total intracranial brain volume). Notably, if we perform an exploratory statistical analysis of the whole cortical mantle using cortical thickness or surface area as measurement of interests (controlling for all covariates), no other brain area showed an association with Observer Injustice Sensitivity that survived whole brain false discovery rate (FDR) correction. This finding further corroborates the results from the VBM 8 analysis.

The article is accompanied by an Excel file containing the main psychometrical and brain measurements used in the reported analyses (see S1 Data File).

## Supporting Information

S1 Data File (XLSX)
